# *Saccharomyces boulardii* CNCM I-745 Modulates the Fecal Bile Acids Metabolism During Antimicrobial Therapy in Healthy Volunteers

**DOI:** 10.3389/fmicb.2019.00336

**Published:** 2019-03-04

**Authors:** Ciaran Patrick Kelly, Caroline Chong Nguyen, Lola Jade Palmieri, Kumar Pallav, Scot E. Dowd, Lydie Humbert, Philippe Seksik, Andre Bado, Benoit Coffin, Dominique Rainteau, Toufic Kabbani, Henri Duboc

**Affiliations:** ^1^Division of Gastroenterology, Department of Medicine, Beth Israel Deaconess Medical Center, Harvard Medical School, Boston, MA, United States; ^2^Inserm UMR1149, DHU Unity – Paris Diderot University, Paris, France; ^3^INSERM U1057, Université Pierre et Marie Curie, Paris, France; ^4^Louis-Mourier Hospital, APHP – University Paris VII, Paris, France; ^5^Molecular Research, Shalltower, TX, United States

**Keywords:** bile acids, probiotics, *Saccharomyces boulardii*, antibiotics, dysbiosis, microbiota

## Abstract

*Saccharomyces boulardii* CNCM I-745 (SB) is a probiotic yeast used to lower the incidence of antibiotic-associated *Clostridium difficile* (*C. difficile*) infection, though its mechanism of action remains unclear. Cholic acid is a primary bile acid, which triggers the germination and promotes the growth of *C. difficile*. The intestinal microbiota transforms primary into secondary bile acids. This study examined (1) the antimicrobial-induced alteration of fecal bile acid content, and (2) whether the concomitant administration of SB influences this transformation. This is an ancillary work from a randomized study, which revealed that SB modulates fecal microbiota dysbiosis during antibiotic treatment. Healthy subjects were randomly assigned to (1) SB only, (2) amoxicillin-clavulanate (AC), (3) SB plus AC, or (4) no treatment. We analyzed fecal concentrations of BA by high performance liquid chromatography/tandem mass spectrometry. Compared to the untreated or the SB-treated groups, AC decreased the percentage of fecal secondary BA significantly (days 3 and 7). When SB and AC were administered concomitantly, this decrease in secondary BA was no longer significant. Following treatment with AC, a significant peak of fecal CA was measured on days 3 and 7, which was prevented by the concomitant administration of SB. AC administered to healthy volunteers altered the microbial transformation of primary BA, decreased secondary BA, and increased CA. The latter was prevented by the concomitant administration of SB and AC, suggesting a potent mechanism protection conferred by SB against post-antimicrobial *C. difficile* infection.

**Clinical Trial Registration:** www.ClinicalTrials.gov, identifier NCT01473368.

## Introduction

The human gut microbiota is a balanced and complex ecosystem, which exerts several physiological functions useful for the host, such as energy storage, digestion, maturation of the immune system and protection against colonization by pathogens (Zoetendal et al., 2006; Neish, 2009; Fujimura et al., 2010; Buffie and Pamer, 2013). Quantitative and qualitative alterations in the gut microbiota are grouped under the term dysbiosis, a deviation away from the intestinal microbial balance. *C*. *difficile* infection is a well-known consequence of antimicrobial-induced dysbiosis, (Abou Chakra et al., 2014) and a major cause of death, disease recurrences and is associated with increased health care costs (O’Brien et al., 2007; Le Monnier et al., 2015; Levy et al., 2015). An estimated 500,000 cases of *C. difficile* infections are responsible for approximately 30,000 deaths per year in the United States (Lessa et al., 2015). Fecal transplantation, a major therapeutic advance, which targets the gut microbiota, is more effective than antimicrobial therapy in the prevention of *C. difficile* infection recurrences (van Nood et al., 2013).

The biotransformation of bile acid is a physiological function fulfilled by intestinal bacteria. These cholesterol derivatives are synthesized by the liver as the primary forms, cholic and CDCA, and conjugated to a taurine or glycine amino acid, before their secretion into the gut lumen. During digestion, they promote the absorption of lipids by forming micelles containing dietary fat, before being reabsorbed in the terminal ileum and return to the liver via the portal circulation, hence forming an enterohepatic cycle. During their passage through the intestinal lumen, the bile acids undergo consecutive metabolic steps performed by the microbiota, including (1) a separation of the amino acids from the sterol nucleus (deconjugation), and (2) the removal of the hydroxyl group on the carbon seven of the sterol nucleus (7-alpha-dehydroxylation).(Hofmann, 1999) In human intestinal disease, dysbiosis has been associated with alteration of both conjugation and transformation of BA (Duboc et al., 2013).

The link between BA and *C. difficile* germination has been known for decades: TCA, a primary bile acid, is regularly used *in vitro* as a powerful germinant of *C. difficile* spores (Wilson et al., 1982; Sorg and Sonenshein, 2008). The unbalance between germinative and non-germinative BA induced by antimicrobial therapy in humans and animals, is suspected to be strongly implicated in primary and recurrent *C. difficile* infections (Theriot et al., 2014; Buffie et al., 2015). In patients suffering from recurrent *C. difficile*, elevated fecal concentrations of CA can be normalized by a fecal microbiota transplantation, probably by restoring the transformation activity of the microbiota (Theriot et al., 2014).

*Saccharomyces boulardii* CNCM I-745 (SB) is a yeast, which can lowers the risk of *C. difficile* colitis caused by antimicrobial therapy (McFarland et al., 1994; Surawicz et al., 2000). Many clinical studies have shown heterogeneous results and inconsistent efficacy, probably depending on the strain causing the infection, type of antibiotics, hospitalized or non-hospitalized patients, or use in primary or secondary prevention (McFarland, 2006; Pozzoni et al., 2012). However, this protective effect has been studied, and several models have been proposed with a view to clarify the mechanisms of action of SB, including interference with bacterial adhesion, inactivation of toxins and other determinants of virulence, and enhancement of mucosal immune function (Castagliuolo et al., 1996; Tasteyre et al., 2002; Chen et al., 2006). A recently published study reported an attenuation of microbiota shifts, including less overgrowth of *Escherichia coli* and a decrease in antimicrobial-associated diarrhea scores, by the addition of SB to AC (Kabbani et al., 2016). The aim of this ancillary study was to compare the effects of SB and AC on the balance between primary and secondary BA in the feces of healthy volunteers.

## Materials and Methods

### Population and Samples

This is a secondary analysis of data collected in an open-label, randomized trial in healthy volunteers between 18 and 65 years of age, who had no history of immunodeficiency or hypersensitivity to yeasts, penicillin or cephalosporin. The details of the main trial have been published previously (Kabbani et al., 2016). All study visits were held at the Harvard Catalyst clinical research center at Beth Israel Deaconess Medical Center, in Boston, MA, United States. Using a computer-generated randomization sequence, subjects were randomly assigned to group 1: SB (*Saccharomyces boulardii* CNCM I-745, Florastor^^®^^; Biocodex Inc.), 500 mg twice daily for 14 days, group 2: AC, 875/125 mg 1 h before meals, twice daily for 7 days, group 3: SB + AC in the same doses and for the same durations as when administered alone, or group 4: no treatment (control group). The 48 volunteers, evenly divided among 4 groups of 12 participants, were instructed to abstain from consuming probiotic supplements, antacids, anti-diarrheal or laxatives, antifungals, other antimicrobials, yogurt or fermented foods containing live yeast for the duration of the study.

The Institutional Review Board of Beth Israel Deaconess Medical Center approved this study and all enrolled volunteers granted their written, informed consent to participate.

### Stool Samples Collections and Analysis

The sampling is summarized in Supplementary Figure S1. Briefly, in the 36 actively treated volunteers, the composition of the fecal microbiota was analyzed in stool samples collected before (at the time of screening and on day 0), during (days 3, 7, 10, and 13), and after (day 21) the administration of treatment. In the control group, the stool samples were collected on days 0, 10, and 21. The samples were frozen at -78.5°C, placed on dry ice inside dedicated containers, shipped within 36 h and processed at INSERM UMR 7203 at Pierre et Marie Curie University. Of the 288 scheduled analyses, a single stool sample from each of three group (SB, AC, and SB + AC) was missing or was not analyzed for BA.

### Extraction of Fecal Bile Acids and Measurements by High Pressure Liquid Chromatography Tandem Mass Spectrometry

Bile acids were measured in feces as described previously (Humbert et al., 2012) and further detailed in the Supplementary Methods. Briefly, the BA were purified by solid phase extraction, and measured by a HPLC MS/MS system. Each peak was identified by comparison to a range of standards that included 28 species of BA (Supplementary Table S1). The quantitative measurements were made, using the Analyst^^®^^ V.1.4.2 software (SCIEX, Framingham, MA, United States). After calibration of the method with weighed mixtures and normalization relative to the internal standard, the measurements were expressed as concentrations or percentages (±SEM) of each bile acid species out of the total bile acid. The BA were grouped by categories (Supplementary Table S1) or expressed individually.

### Statistical Analyses

The measurements are presented as means ± SEM. Paired *t*-tests were used to compare within-groups measurements made over time. Comparisons among multiple groups were made at each sampling time, using one-way ANOVA, followed by Tukey’s multiple comparison test, comparing groups 1, 2, and 3. Group 4 was excluded from the multiple comparison because of an insufficient number of measurements. The outliers were suppressed after statistical confirmation. *P*-values <0.05 were considered statistically significant. All analyses were performed using the Prism 6^1^ Graphpad software (GraphPad Software, Inc., La Jolla, CA, United States).

## Results

**FIGURE 1:**
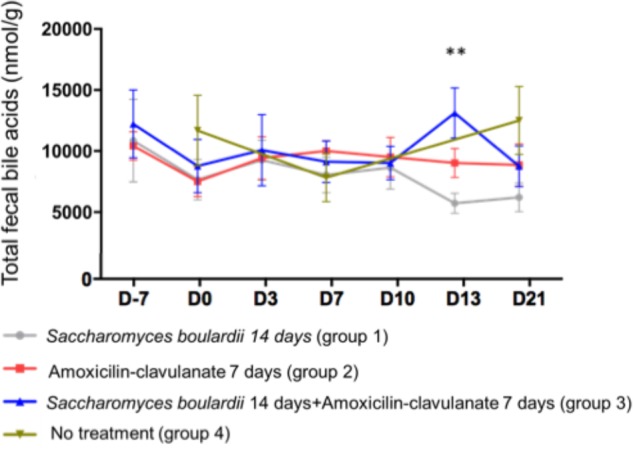
Mean ( ± SEM) total fecal bile acid concentration in healthy volunteers. ^∗∗^*p* < 0.01 (one-way ANOVA).

We analyzed BA in the feces of 4 groups of 12 healthy subjects. Group 1 was treated with SB CNCM I-745 for 14 days; group 2 received AC for 7 days; group 3 was treated with SB for 14 days + AC for 7 days; group 4 received no treatment. Regarding the total fecal BA concentration, no significant change from baseline was observed at any time point, except for a single increase on day 13 in group 3 (treated with SB + AC), which had returned to baseline by day 21 (Figure 1).

**FIGURE 2:**
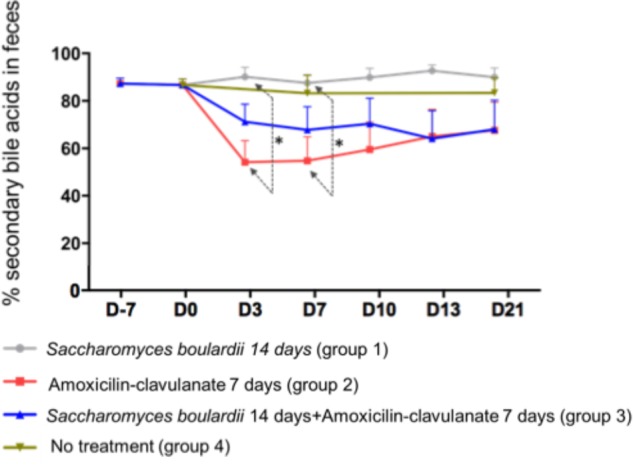
Comparison of percentages of secondary fecal bile acid using one-way ANOVA followed by multiple comparison analysis (Tukey’s test) at each sampling point. Data from samples before any treatment (D10 and D0) were pooled on the figure. ^∗^*p* = 0.03.

**FIGURE 3:**
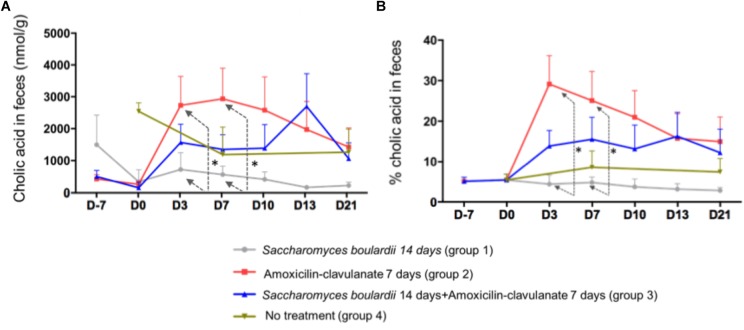
**(A)** Comparison of fecal concentrations of cholic acid using one-way ANOVA followed by multiple comparison analysis (Tukey’s test) at each sampling point. D3, ^∗^*p* = 0.013; D7, ^∗^*p* = 0.037 (one-way ANOVA between the groups SB, AC, and SB+AC). **(B)** Comparison of percentages of CA using one-way ANOVA followed by multiple comparison analysis (Tukey’s test) at each sampling point. Data from samples before any treatment (D10 and D0) were pooled on the figure D3, ^∗^*p* = 0.0037; D7, ^∗^*p* = 0.035.

Analysis of the composition of the fecal pool over time revealed stable percentages of secondary BA (Figure 2), and no significant differences on days 2, 4, and 7 between healthy volunteers and patients treated with SB only. We then looked for an the effect of antimicrobials administration on secondary BA, and CA. The administration of AC lowered the proportions of secondary BA significantly on days 3 (-33.1% from baseline), 7 (-33.2% from baseline), and 10 (-28.5%), followed by a slow return to baseline (Figure 2, detail in Supplementary Figure S2). In mirror, between baseline and day 3, antimicrobial therapy increased the concentration of fecal CA in group 2 from 260 ± 110 to 2,733 ± 913 nmol/g of dried stool (*p* < 0.05), followed by a gradual return toward baseline between day 7 and day 21 (Figure 3A, detail in Supplementary Figure S2). The same kinetic profile was observed using the percentages of CA (Figure 3B, detail in Supplementary Figure S2).

We then looked if the co-administration of SB modulates theses changes induced antimicrobials on BA metabolism. By one way analysis of variance (ANOVA), the percentages of secondary BA in feces varied significantly among groups 1 (SB), 2 (AC), and 3 (AC + SB) on days 3 and 7 (Figure 2). A multiple comparison analysis (Tukey’s test), revealed a significantly lower percentage of secondary fecal BA in the AC-treated than in the SB + AC-treated group on days 3 and 7 (Figure 2).

The combined administration of SB with AC had also an effect on CA (Figure 3A,B) at D3 and 7, the AC-treated volunteers had a significant and rapid increase in CA percentage compared to SB-treated group. This peak was also observed using CA concentration. However, this increase was no longer significant using a combined administration of AC + SB.

## Discussion

In this study we analyzed fecal bile salts in samples from a prospective, randomized, controlled, open-label, human clinical trial in healthy volunteers (Kabbani et al., 2016). We first observed that the administration of antimicrobials profoundly decreases the transformation of bile acid by the microbiota, a decrease that spontaneously returns to baseline over time after the completion of antibiotic administration. Concordant increases were observed in the fecal concentrations and proportions of CA. We also found that the lone administration of the probiotic yeast SB CNCM I-745 did not influence the bile acid metabolism. Interestingly, when administered during and after AC, SB prevented, in a prolonged manner, the decrease in the transformation of BA, and the increase in fecal CA.

We used high-performance liquid chromatography (HPLC) coupled to tandem mass spectrometry (MS/MS), a validated and accurate method of bile acid measurement in feces (Humbert et al., 2012). The effect of SB on the transformation of BA, analyzed over consecutive days, was sustained, a more relevant observation than an isolated difference at a single time point. This effect mediated by a probiotic raises the issue of a host-dependent effect driven by interactions with the gut mucosa, vs. an intra-luminal effect linked to the microbiota. The study of healthy volunteers instead of patients suffering from gastro-intestinal disorders, with an antimicrobial-induced dysbiosis, was a means of identifying a mechanism probably more dependent on the microbiome and SB than on the host.

In this study, antibiotic administration was used as an experimental model of dysbiosis. Similar effects of fecal dysbiosis on bile acid metabolism have been described during IBD, consisting of a decrease in the transformation of primary BA, and the de-conjugation of amino-conjugated bile acid in feces (Duboc et al., 2013). In this study, the fecal percentages and concentrations of amino-conjugated bile acid were similar over time and among groups (representing less than 4% of the fecal bile acid pool, Supplementary Figure S2). This could be explained by a greater redundancy of deconjugating bacteria, a more resilient ecosystem regarding the deconjugation function, or both. A less prominent and less specific dysbiosis than in patients suffering from acute IBD, might also explain this difference.

We hypothesize a mechanism by which SB protects the metabolism of BA, based on the initial observations made by Kabbani et al. (2016), who analyzed the composition of the intestinal microbiome in the same fecal samples of these volunteers. They found that the oral administration of SB to healthy subjects had no effect on the composition of the core microbiome of their fecal samples. However, as previously described, antimicrobials cause profound shifts to the core microbiome, with a return toward baseline status following treatment (De La Cochetière et al., 2005; Dethlefsen et al., 2008; Jernberg et al., 2013). We think theses shifts could direct the microbiota toward a lower capacity to perform bile acid transformation, explaining the peak of CA. Here, the concomitant administration of SB and AC partially prevents the antibiotic-induced dysbiosis, which could explain why the transformation of BA is less impaired by antimicrobials when SB is orally taken. However, analysis of the fecal composition by pyrosequencing cannot quantify or precisely reflect the microbial functions preserved by the probiotic treatment. The transformation of bile acid is a multi-enzymatic process assumed by many bacteria, a function difficult to evaluate by molecular analytical methods.

The transformation of primary to secondary BA is a dehydroxylation effected by the microbiota.(Ridlon et al., 2006) Primary BA and glycine act as germinants for *C. difficile* spores (Wilson et al., 1982; Sorg and Sonenshein, 2008; Francis et al., 2013). Therefore, a balance between primary and secondary BA modified by antimicrobial therapy and an increase in CA in particular, might contribute to the germination and growth of *C. difficile*.

*Saccharomyces boulardii* might prevent *C. difficile* infections by attenuating the changes in bile acid transformation, such as the presence of CA in feces. This is consistent with observations made in previous studies of the transformation of fecal microbiota. Patients suffering from recurrent *C. difficile* infections presented with high concentrations of fecal CA, (Allegretti et al., 2016) and the transformation of colonic microbiota, by fecal microbiota transplantation, lowered and restored the normal concentrations of fecal CA (Weingarden et al., 2014).

We hypothesize that SB lowers the incidence of antimicrobial-associated *C. difficile* infections by attenuating antibiotic-induced elevations in colonic CA. This primary bile acid, and other CA derivatives, including taurocholic and glycocholic acids, trigger the germination of *C. difficile* spores *in vitro* via the germinant receptor, CspC.(Francis et al., 2013) On the other hand, secondary BA produced by the gut microbiota, inhibit spore germination *in vitro* (Sorg and Sonenshein, 2008, 2010; Theriot et al., 2014; Weingarden et al., 2016). BA are also implicated in bacterial outgrowth.

In conclusion, the composition of fecal BA results from an amalgam of host bile acid production and microbiome-driven metabolism, that can be disturbed by antibiotics administration in healthy volunteers. Further studies are needed in patients experimenting CDI to confirm if these changes in the fecal BA composition are clinically implicated in the pathophysiology of the infection.

## Data Availability

The raw data supporting the conclusions of this manuscript will be made available by the authors, without undue reservation, to any qualified researcher.

## Author Contributions

CK, KP, SD, AB, DR, TK, and HD contributed to conception and design of the study. LP, HD, CCN, LH, PS, and DR contributed to the acquisition of data, analysis and interpretation of data. LP, CCN, and HD performed the statistical analysis. LP, HD, and CK wrote the first draft of the manuscript. BC and AB wrote sections of the manuscript. SD, DR, and BC made critical revision of the manuscript for important intellectual content. All authors contributed to manuscript revision, read and approved the submitted version.

## Conflict of Interest Statement

HD has worked as a scientific advisor for Ipsen laboratory and Biocodex laboratory. CK has acted as a speaker for Biocodex at educational events. The remaining authors declare that the research was conducted in the absence of any commercial or financial relationships that could be construed as a potential conflict of interest.

## Footnotes

1 https://graphpad.com/quickcalcs/Grubbs1.cfm

## Supplementary Material

FIGURE S1 First panel: the treatment received in the four groups of healthy subjects. Second panel: the number of samples available at each time of sampling.

FIGURE S2 The 2 graphs on the left shows the absence of difference in fecal conjugated bile acids, both in concentration and in %. The upper right figure shows the drop in secondary bile acids % in the antibiotics group, and the lower right figures shows the peak of CA concentration in the antibiotics group.

TABLE S1 List of the 28 bile acids species measured in feces.

## Abbreviations

AC: amoxicillin-clavulanate
BA: bile acids
*C. difficile*: *Clostridium difficile*
CA: cholic acid
CDCA: chenodeoxycholic acid
DCA: deoxycholic acid
IBD: inflammatory bowel disease
LCA: litocholic acid
SB: *Saccharomyces boulardii*
TCA: taurocholic acid
