# Supplementary figures and images for: Saccharomyces boulardii CNCM I-745 Modulates the Fecal Bile Acids Metabolism During Antimicrobial Therapy in Healthy Volunteers
Source: Front Microbiol. 2019 Mar 4;10:336. doi: 10.3389/fmicb.2019.00336 (PMC6407479; doi:10.3389/fmicb.2019.00336)

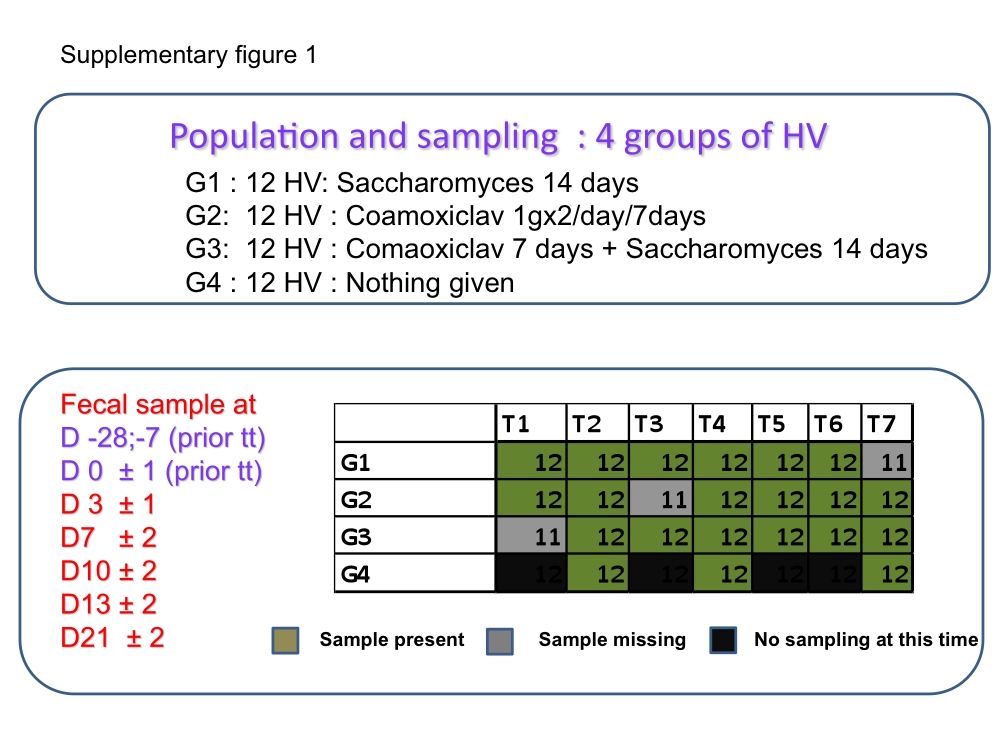

Supplement: FIGURE S1 — First panel: the treatment received in the four groups of healthy subjects. Second panel: the number of samples available at each time of sampling. [file Image_1.TIF]

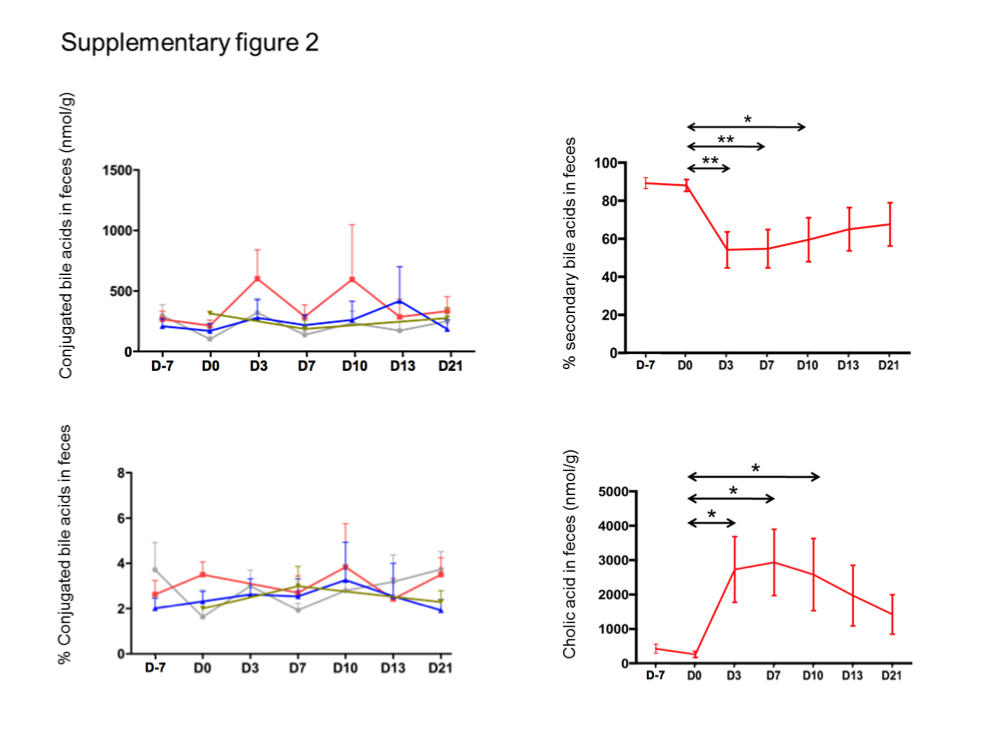

Supplement: FIGURE S2 — The 2 graphs on the left shows the absence of difference in fecal conjugated bile acids, both in concentration and in %. The upper right figure shows the drop in secondary bile acids % in the antibiotics group, and the lower right figures shows the peak of CA concentration in the antibiotics group. [file Image_2.TIF]
