# Supplementary material for: Saccharomyces boulardii CNCM I-745 Modulates the Fecal Bile Acids Metabolism During Antimicrobial Therapy in Healthy Volunteers
Source: Front Microbiol. 2019 Mar 4;10:336. doi: 10.3389/fmicb.2019.00336 (PMC6407479; doi:10.3389/fmicb.2019.00336)
Supplement: TABLE S1 — List of the 28 bile acids species measured in feces. [file Table_1.pdf]

Supplementary methods end Figures

Bile acids quantitation in feces

All chemicals and solvents were of the highest purity. Cholic acid, deoxycholic acid, chenodeoxycholic acid, ursodeoxycholic acid, lithocholic acid, hyocholic acid and corresponding glyco- and tauro-derivatives were obtained from Sigma-Aldrich. The 3-sulphate derivatives were a generous gift from Dr J Goto (Niigata University of Pharmacy and Applied Life Sciences, Niigata, Japan) and the 23-nor-5 β-cholanoic acid-3α, 12α-diol was purchased from Steraloids, Inc. 3-sulpholithocholic acid was synthesized in our laboratory (ENS, Paris) using a previously described method,[27] and further characterized by nuclear magnetic resonance and mass spectrometry. Acetic acid, ammonium carbonate and ammonium acetate were purchased from Sigma-Aldrich.

Standard stock solutions were prepared in methanol at a concentration of 1 mg/ml and stored in a sealed container at −20°C. The stock solutions were pooled and diluted to obtain mixed-calibration bile acid solutions, ranging from 31.3 mg/ml to 31.3 ng/ml.

Sample preparation: Two µl of standard internal solution (23-nor-5β-cholanoic acid-3α, 12α-diol at 1 mg/ml) was added to 0.1 g of fecal lyophilized samples using a Thermo Savant Speedvac (SPD 111V) coupled to a cooled vapor trap (RTV400). The bile acids were released from the binding protein by the addition of 0.4 M ammonium carbonate, at a concentration of 4 ml ammonium carbonate per 1 ml of sample, and incubated for 30 min at 60°C. For the fecal samples, 2 ml of NaOH (0.1 M) was added and incubated for 1 h at 60°C before the addition of 4 ml of water.[28] The solution was homogenized by two 30-s runs in an Ultra-Turrax® disperser (IMLAB).

Pre-analysis clean-up procedure: centrifugation of the sample at 20,000×g for 20 min, followed by solid-phase extraction using reversed-phase silica cartridges. These reversed-phase, 100-mg Chromabond C18 cartridges (Macherey-Nagel) were preconditioned with 5 ml

of methanol and 5 ml of water in succession. The samples were then loaded on the cartridge, and the subsequent elution steps were processed using a vacuum manifold. The cartridge was rinsed with 20 ml of water, followed by 10 ml of hexane, then rinsed a second time with 20 ml of water. The bile acids were finally eluted and collected by methanol eluate. The methanol was evaporated under nitrogen at 50°C, and the residue was re-suspended in 150 µl of methanol, 5 ml of which was injected into the HPLC MS/MS system.

HPLC MS/MS analysis: The bile acids were separated as a function of polarity, using a 250 mm long x 3.2 mm in diameter Pinnacle II C18 analytical column (Restek), 5-mm silica particle (Restek)) fitted on an Agilent 1100 HPLC binary pump (Agilent Technologies). The transfer line from the autosampler (Agilent) and column was maintained at 35°C. The 0.3–0.5 ml/min flow rate was increased during the elution protocol. The mobile phase was composed of a 15-mM mixture of ammonium acetate at pH 5.3 and methanol. The HPLC was coupled in series with the turbo ion-spray source of the QTRAP 2000 tandem mass spectrometer (Applied Biosystems/MDS SCIEX). Electrospray ionization was performed in the negative mode, with nitrogen as the nebulizer gas. The temperature of the evaporation gas was set at 400°C. The ion-spray, declustering and entrance potentials were set at −4,500 V, −60 V and −10 V, respectively. The collision-induced dissociation was achieved in a Q2 collision cell under various voltage potentials, depending on conjugation, and the MS/MS detection was operated with unit/unit resolution in the multiple-reaction monitoring mode. The dwell time of the ion trap was set at 70 ms for each transition.

Data acquisition: the data were extracted using Analyst V.1.4.2 software. Multiple-reaction monitoring at low collision energy focuses on transition reactions from precursor ions to product ions after the cleavage of taurine, glycine and sulphate fragments. For glycine-conjugated bile acids, m/z 432, 448 and 464 were selected as the precursor ions, and m/z 74 was selected as the product ion. For taurine-conjugated bile acids, m/z 482, 498 and 514 were

selected as the precursor ions, and m/z 80 was selected as the product ion. For sulpho-conjugated bile acids, the HSO4 sulphuric anion (m/z 97) from the sulphate moiety was selected as the product ion. For unconjugated bile acids, m/z 375, 391 and 407 were selected as the precursor and the product ions, as no fragmentation could be identified at the low collision energy used. In addition, m/z 377 was selected for the internal standard (23-nor-5 β-cholanoic acid-3α, 12α-diol). The bile acid measurement was expressed as the percentage ± SEM of each specific bile acid, out of the total bile acids, after calibration of the method, with weighed mixtures and normalization relative to the internal standard (nordeoxycholic acid).

Supplementary table 1: List of the 28 bile acids species measured in feces

Bile acids: Primary	Bile acids: Secondary	Ursodeoxycholic acids
Cholic acid	Deoxycholic acid	Ursodeoxycholic acid
Tauro-Cholic acid	Tauro-Deoxycholic acid	Tauro-Ursodeoxycholic acid
Glyco-Cholic acid	Glyco-Deoxycholic acid	Glyco-Ursodeoxycholic acid
Cholic acid-3S	Deoxycholic acid-3S	Ursodeoxycholic acid-3S
Chenodeoxycholic acid	Lithocholic acid	Tauro-Ursodeoxycholic acid-3S
Tauro-Chenodeoxycholic acid	Tauro-Lithocholic acid	Glyco-Ursodeoxycholic acid-3S
Glyco-Chenodeoxycholic acid	Glyco-Lithocholic acid	
Chenodeoxycholic acid-3S	Lithocholic acid-3S	
Hyocholic acid	Tauro-Lithocholic acid-3S	
Muricholic acid	Glyco-Lithocholic acid-3S	
	Hyodeoxycholic acid	
	Tauro-Hyodeoxycholic acid	

3S suffix indicates sulfation on carbon 3
